# Supplementary material for: Limited Expression of Nrf2 in Neurons Across the Central Nervous System
Source: bioRxiv. 2023 May 9:2023.05.09.540014. Preprint. [Version 1] doi: 10.1101/2023.05.09.540014 (PMC10197674; doi:10.1101/2023.05.09.540014)
Supplement: Supplement 1 [file NIHPP2023.05.09.540014v1-supplement-1.pdf]

bioRxiv preprint doi: https://doi.org/10.1101/2023.05.09.540014; this version posted May 9, 2023. The copyright holder for this preprint (which was not certified by peer review) is the author/funder, who has granted bioRxiv a license to display the preprint in perpetuity. It is made available under aCC-BY 4.0 International license.

Supplementary Figure Legends

Figure S1. Dot plot summarizing aggregate scRNA-seq analysis of *Nrf2* expression in the adult mouse brain. As in Figure 1, cell types are separated based on classification as neurons or support cells, and median expression for each class is indicated with a black horizontal line. Data are from (Saunders et al., 2018).

Figure S2. (A - C) Aggregate snRNA-seq analysis of *NRF2* expression in the human brain, with expression levels of **(A)** the neuronal marker *RBFOX2*, **(B)** the astrocyte marker *AQP4*, and **(C)** the astrocyte marker *SLC1A3* overlaid on each cell type dot, same as in Figure 1B. Data are from (Bhaduri et al., 2021). **(D)** Principal component analysis of aggregate cell type-summarized expression data, based on published cell type assignments (Bhaduri et al., 2021). Neuronal cell populations (blue and green dots) largely cluster separately from the other cell populations of the brain (i.e., support cells). Consistent with the patterns in panels A-C and Figure 1C, the three outlier neuron cell types with higher NRF2 expression (Neuron_11, Neuron_36, Neuron_64; marked with blue arrows) cluster separate from the other neurons and closer to support cells.

Figure S3. Aggregate analysis of *Nrf2* expression in the developing mouse brain. Same as Figure 1C, only with y-axis (*Nrf2* expression level) on a $\log_{10}$ scale rather than a linear scale. To display this data properly on a $\log_{10}$ scale, a pseudocount 1e-4 was added to each cell to avoid zeros.

Figure S4. Heatmap of the aggregate snATAC-seq signal at the *Nrf2* (i.e., *Nfe2l2*) locus across cell types of the mouse brain. Same as Figure 2A, only with cell type abbreviations labeled for each row of heatmap; abbreviations are as described in (Li et al., 2021).

Figure S5. Hierarchical clustering of the aggregate snATAC-seq signal at the *Nrf2*/*Nfe2l2* gene locus. Clustering was based on the signal from the *Nrf2*/*Nfe2l2* gene body and 5 kilobases upstream of the transcription start site. The two major clusters are highlighted yellow and purple. Support cell types are indicated in red text and with red boxes to the right of the heatmap; all non-red cell types are classified as neurons.

Figure S6. Dot plot summarizing aggregate analysis of *Keap1* expression in the developing mouse brain. As in Figure 1C, cell types are separated based on their classification as neurons, neuroblasts, neural tube cells, neural crest cells, and support cells as indicated. Dot color represents the median embryonic stage (e7 through e18) for the population of cells assigned to each cell type. Data are from (La Manno et al., 2021).

Figure S7. Dot plot summarizing aggregate scRNA-seq analysis of Nrf2 target gene expression in the adolescent mouse brain. Similar to Figure 1A, only four direct Nrf2 target genes (*Slc3a2*, *Slc7a11*, *Nqo1*, and *Prdx1*) are represented. Data are from (Zeisel et al., 2018).

bioRxiv preprint doi: https://doi.org/10.1101/2023.05.09.540014; this version posted May 9, 2023. The copyright holder for this preprint (which was not certified by peer review) is the author/funder, who has granted bioRxiv a license to display the preprint in perpetuity. It is made available under aCC-BY 4.0 International license.

Supplementary Figures

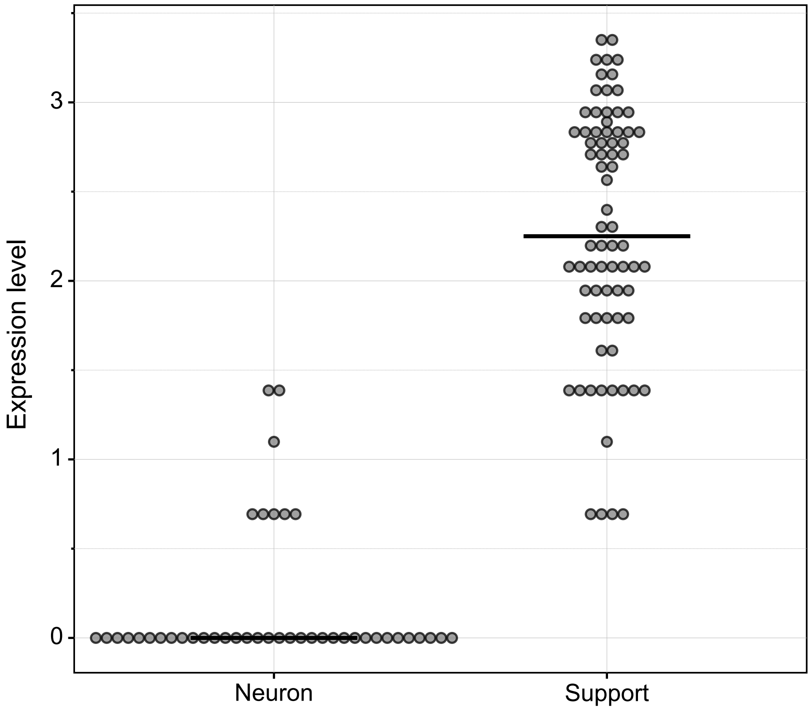

Levings et al., Figure S1

bioRxiv preprint doi: https://doi.org/10.1101/2023.05.09.540014; this version posted May 9, 2023. The copyright holder for this preprint (which was not certified by peer review) is the author/funder, who has granted bioRxiv a license to display the preprint in perpetuity. It is made available under aCC-BY 4.0 International license.

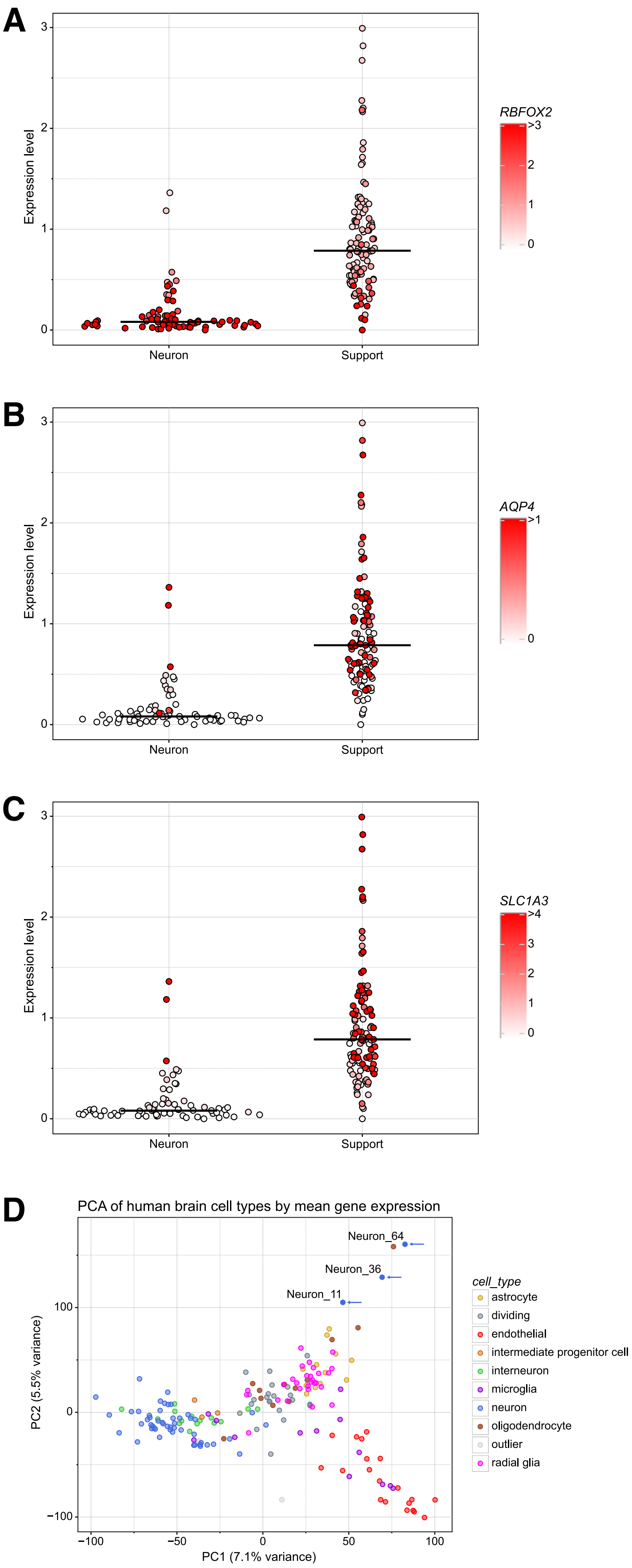

Levings et al., Figure S2

bioRxiv preprint doi: https://doi.org/10.1101/2023.05.09.540014; this version posted May 9, 2023. The copyright holder for this preprint (which was not certified by peer review) is the author/funder, who has granted bioRxiv a license to display the preprint in perpetuity. It is made available under aCC-BY 4.0 International license.

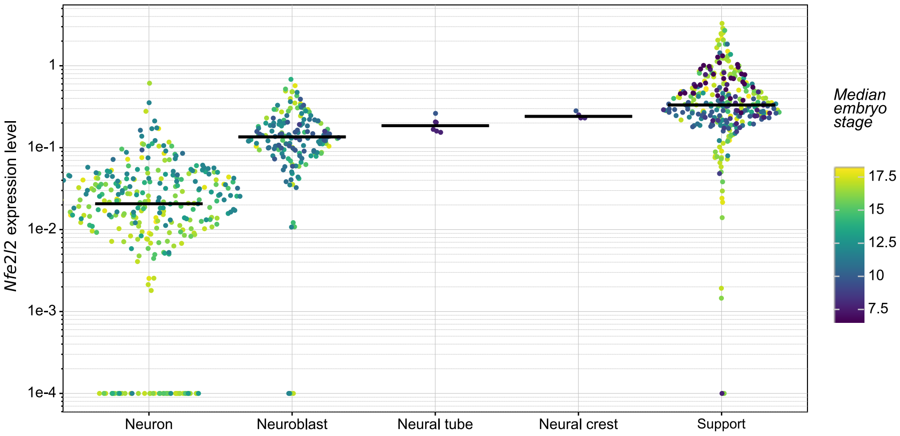

Levings et al., Figure S3

bioRxiv preprint doi: https://doi.org/10.1101/2023.05.09.540014; this version posted May 9, 2023. The copyright holder for this preprint (which was not certified by peer review) is the author/funder, who has granted bioRxiv a license to display the preprint in perpetuity. It is made available under aCC-BY 4.0 International license.

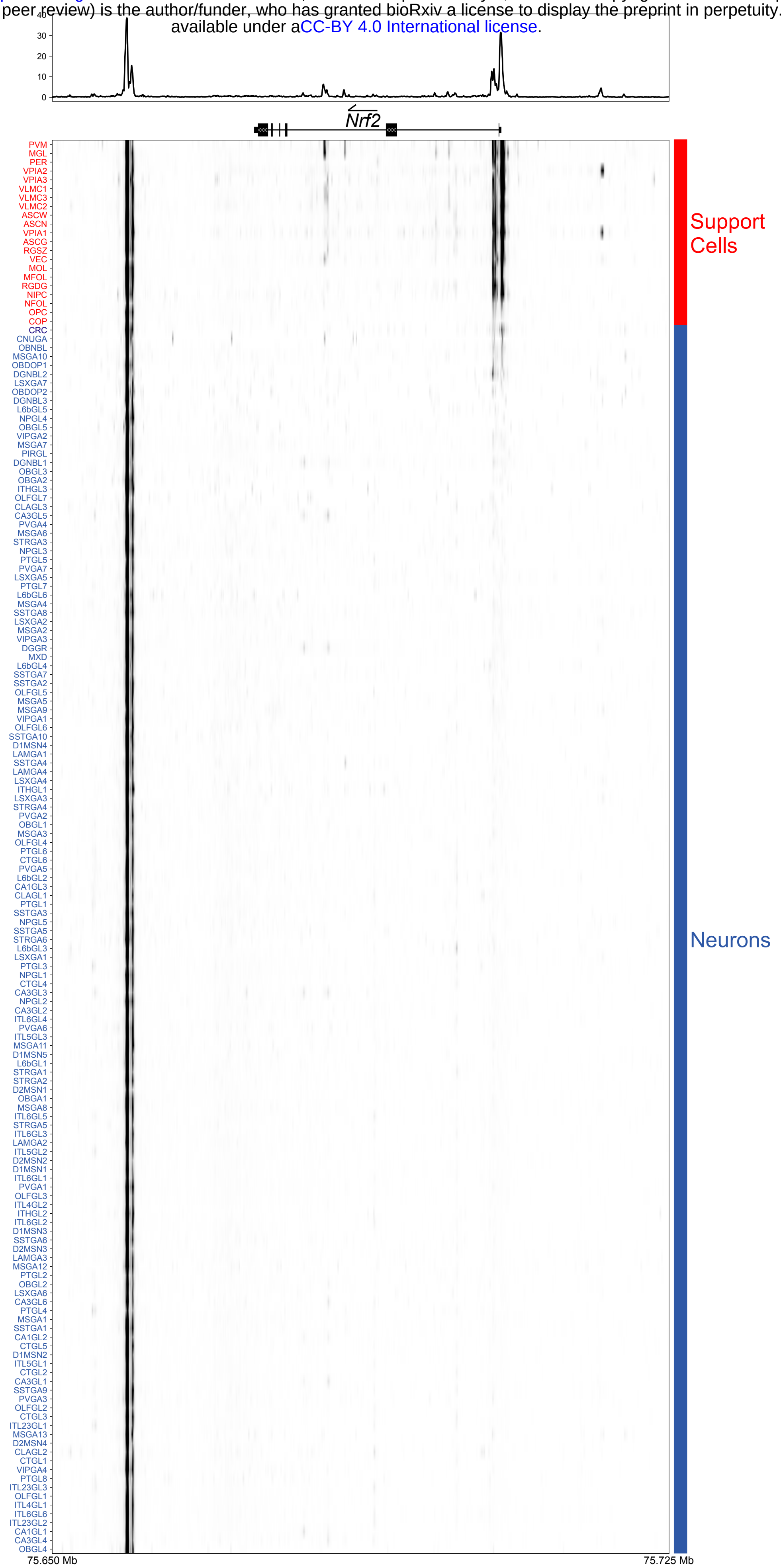

Levings et al., Figure S4

bioRxiv preprint doi: https://doi.org/10.1101/2023.05.09.540014; this version posted May 9, 2023. The copyright holder for this preprint (which was not certified by peer review) is the author/funder, who has granted bioRxiv a license to display the preprint in perpetuity. It is made available under aCC-BY 4.0 International license.

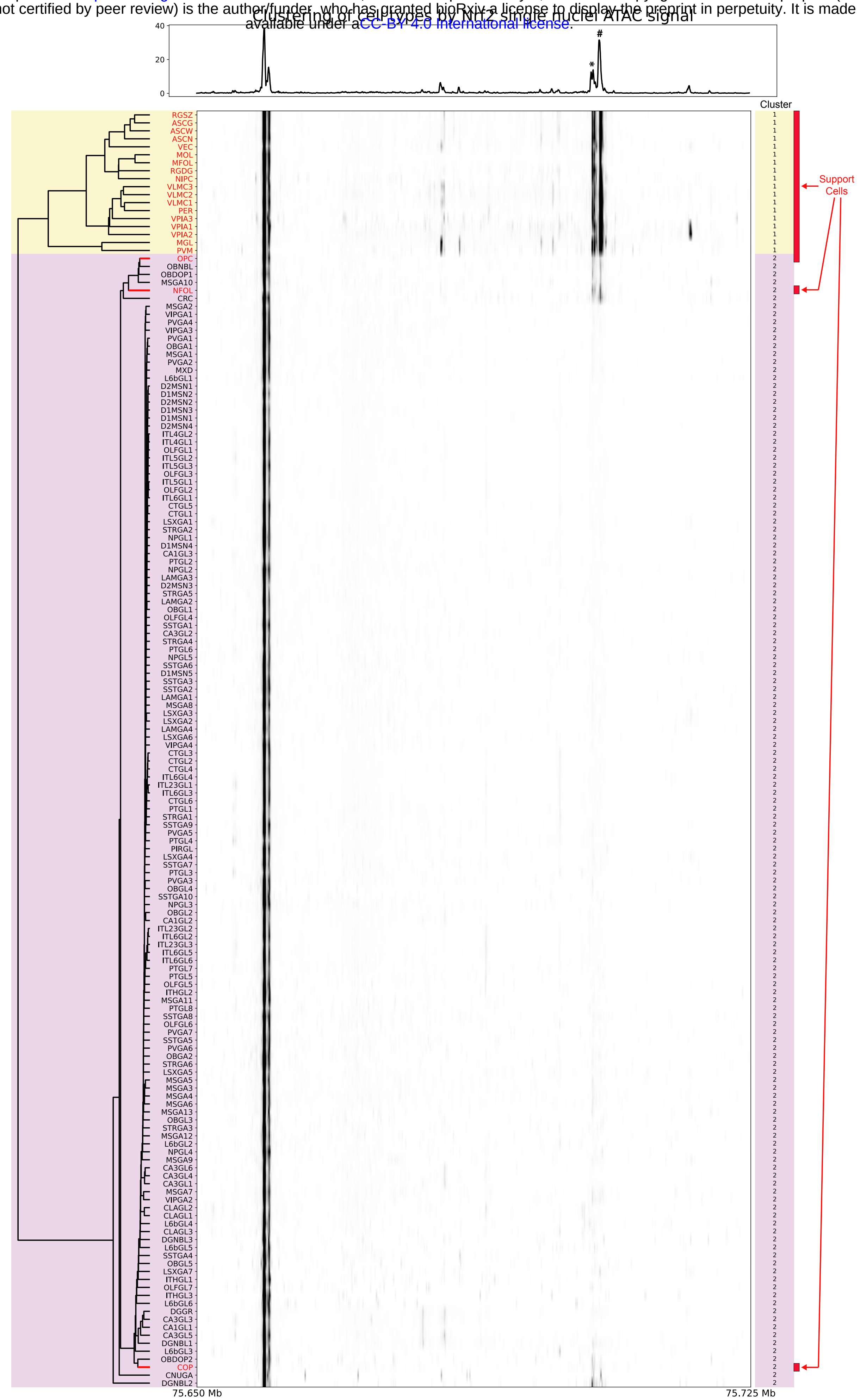

Levings et al., Figure S5

bioRxiv preprint doi: https://doi.org/10.1101/2023.05.09.540014; this version posted May 9, 2023. The copyright holder for this preprint (which was not certified by peer review) is the author/funder, who has granted bioRxiv a license to display the preprint in perpetuity. It is made available under aCC-BY 4.0 International license.

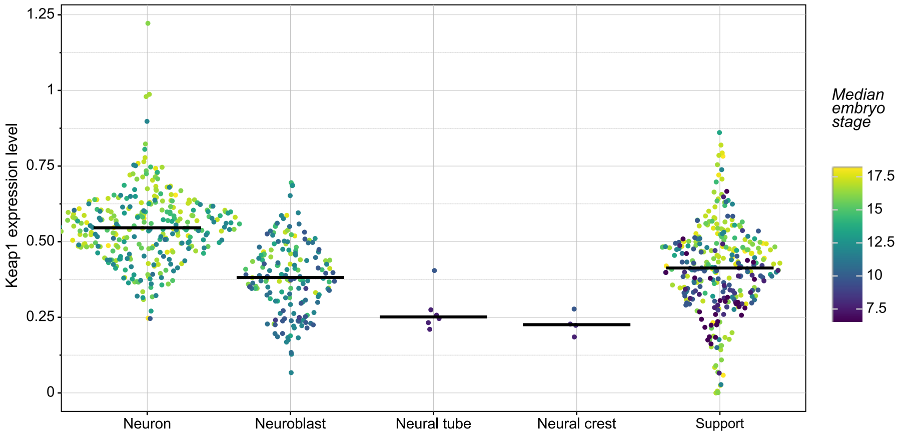

Levings et al., Figure S6

bioRxiv preprint doi: https://doi.org/10.1101/2023.05.09.540014; this version posted May 9, 2023. The copyright holder for this preprint (which was not certified by peer review) is the author/funder, who has granted bioRxiv a license to display the preprint in perpetuity. It is made available under aCC-BY 4.0 International license.

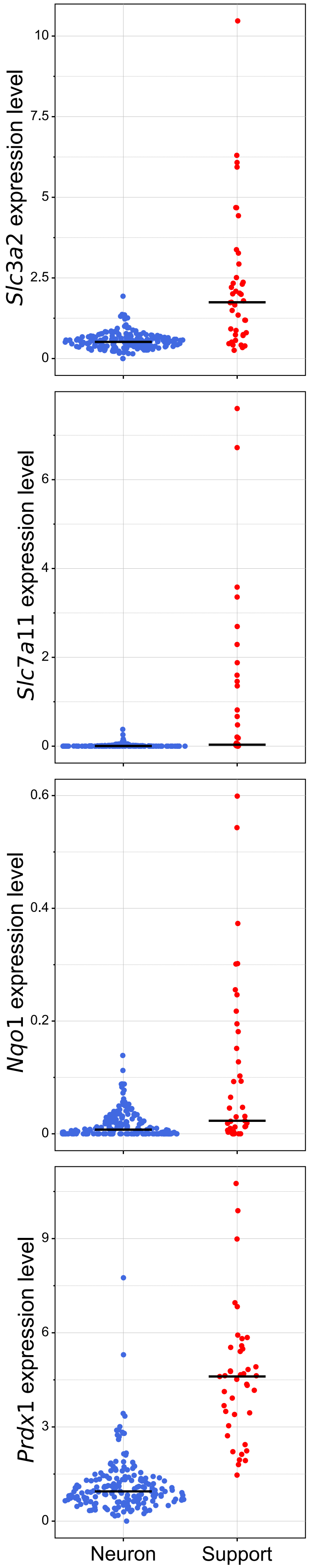

Levings et al., Figure S7
